# Heart Rate Variability Analysis in Congestive Heart Failure: The Need for Standardized Assessment Protocols

**DOI:** 10.31083/RCM36321

**Published:** 2025-05-26

**Authors:** Monika Míková, David Pospíšil, Jan Řehoř, Marek Malik

**Affiliations:** ^1^Department of Internal Medicine and Cardiology, University Hospital Brno and Faculty of Medicine of Masaryk University, 625 00 Brno, Czech Republic; ^2^National Heart and Lung Institute, Imperial College, ICTEM Building, Hammersmith Campus, W12 0NN London, UK

**Keywords:** congestive heart failure, heart rate variability, time-domain methods, spectral analysis, risk prediction

## Abstract

Heart rate variability (HRV) analysis is a noninvasive tool that allows cardiac autonomic control to be assessed. Numerous studies have reported HRV measurements, related changes, and clinical implications for heart failure patients. This review evaluates HRV characteristics in congestive heart failure (CHF), focusing on different recording durations and the diagnostic and prognostic values using HRV measurements. The recording durations are classified as (a) ultra short-term (substantially shorter than 5 minutes), (b) short-term (5 minutes), and (c) long-term (nominal 24 hours). This review of HRV diagnostic and prognostic significance in CHF focuses on time- and frequency-domain HRV measures that have previously been extensively studied. Reported studies document that HRV is lowered in CHF patients, whereas HRV increases may indicate disease improvement, *e.g.*, in CHF patients undergoing cardiac resynchronization therapy. Reduced HRV has consistently been found to be associated with all-cause mortality in CHF patients. However, different thresholds of long-term HRV indices have been proposed as mortality predictors; meanwhile, findings related to the prediction of other cardiac events, including sudden cardiac death, remain inconsistent. HRV is reduced in CHF patients, but the use of HRV as a risk factor remains controversial, with no established cut-off values. HRV does not provide a clinically useful prediction of sudden cardiac death or other cardiac events in CHF patients. Thus, we advocate standardization of investigative protocols based on the existing time- and frequency-domain HRV indices rather than further developing more complex methods. Short-term recordings are preferable for clinical application and measurement reproducibility; thus, future investigations should focus on the following key questions:

1. How to design standardized short HRV tests suitable for outpatient settings?

2. Which HRV indices should be preferred, and what are their optimal prognostic thresholds?

3. How to standardize HRV assessment conditions to minimize external influences?

## 1. Introduction

### 1.1 Congestive Heart Failure

Congestive heart failure (CHF) became a cardiovascular epidemic in the 21st century [1]. In developed countries, 1–2% of the population suffer from CHF which is present in approximately 26 million people worldwide [2]. CHF is characterized by blood volume and/or blood pressure overload arising from abnormalities in cardiac hemodynamic function [3, 4]. CHF may result from systolic dysfunction when impaired myocardial contractility leads to a reduced left ventricular ejection fraction, diastolic dysfunction marked by impaired relaxation and insufficient filling of the left ventricle, or a combination of systolic and diastolic dysfunction. Typical CHF symptoms include reduced exercise tolerance, increased fatigability, chronic weakness, dyspnea, orthopnea, and nocturia. In more advanced cases, patients experience peripheral edema and palpitations, often presenting as rapid or irregular cardiac periods. CHF is predominantly a consequence of prior cardiovascular disorders, including ischemic heart disease, dilated or hypertrophic cardiomyopathy, hypertension, and heart valve disease [5]. As CHF progresses, anatomical, functional, neurohormonal, and electrophysiological alterations increase mortality [6]. The failing cardiac hemodynamic function also causes secondary autonomic nervous system (ANS) changes.

### 1.2 Autonomic Nervous System Regulation

Balancing the sympathetic and parasympathetic ANS branches is crucial in regulating cardiovascular function. A complex interplay between ANS branches exists in healthy subjects and patients with different abnormalities and pathologies. The parasympathetic system reacts faster than the sympathetic system, which has known advantages for assessing ANS regulation and reflexes [7, 8]. The sympathovagal balance is often disrupted in CHF patients since CHF is associated with chronic sympathetic overactivity due to reduced cardiac output and lower tissue perfusion [9]. Consequently, CHF is often accompanied by reduced parasympathetic (vagal) activity [10], which lowers the ability of ANS to modulate heart rate and maintain cardiovascular homeostasis.

The cardiovascular system involves complex interconnected feedback mechanisms. The feedback maintenance leads to various cardiovascular fluctuations that might be detected and numerically characterized. These fluctuations result from continuous cardiovascular function adjustments, which respond to internal and external challenges and maintain organism stability. Heart rate and blood pressure are modulated in real time by the ANS, hormonal signals, and baroreflex activity [11]. These regulatory mechanisms respond to the requirements of the organism in variable situations, including rest, exercise, and stress. Moreover, the ongoing adjustment of cardiac periodicity leads to measurable variability expressed by heart rate variability (HRV), blood pressure variability, and other characteristics that allow numerical quantification. Ultimately, these measurable fluctuations reflect the ability of the cardiovascular system to adapt when responding to intrinsic demands and environmental stresses [12, 13, 14].

### 1.3 Heart Rate Variability

HRV is a recognized method to assess cardiac autonomic modulations. HRV is based on the fluctuations in the intervals between consecutive QRS complexes (waveform segment composed of the Q, R, and S deflections, representing the electrical depolarization of the ventricles) of normal sinus nodal origin, *i.e.*, the so-called normal-to-normal (NN) intervals [15, 16, 17, 18, 19]. Numerous intrinsic factors, such as respiration frequency, body temperature, food intake, sleep and sleep stages, electrolyte levels, humoral control, and sympathovagal balance, regulate sinus rhythm periodicity. HRV also responds to external factors, including daylight, weather, pollution, and perception of threat or tranquil safety. Consequently, HRV is a complex indicator influenced by autonomic modulation and physiological, pathological, and environmental factors. In addition, different pharmaceuticals and recreational substances are known to affect heart rate and impact ANS [20], which is also reflected in HRV changes [21, 22].

Clinically, HRV is useful for assessing autonomic regulation in conditions such as sub-acute myocardial infarction, diabetic autonomic neuropathy, and acute or chronic stress [23, 24, 25, 26, 27]. In cardiac patients, HRV assessment also provides risk characterization and prognosis and allows patient monitoring during disease progression. However, broader clinical applications are limited by measurement variability, external influences, and the lack of standardized normality thresholds [28].

Over the past 40 years, many publications have focused on HRV assessment in CHF patients; thus, HRV characteristics are now well-known to be significantly different in CHF patients compared to healthy subjects [29, 30]: HRV is depressed in CHF patients [16, 31, 32, 33]. This reduction is mainly related to increased sympathetic activity, decreased vagal tone, and depressed baroreceptor responsiveness [34, 35]. Since HRV is a marker of sympathetic and parasympathetic influence on heart rate modulations [36], it has also been proposed as a stratification marker of cardiovascular mortality risk [37, 38, 39], CHF severity [40], and CHF progression [33]. However, the exact predictive thresholds of HRV in CHF patients are difficult to standardize as a variety of HRV parameters have been derived from a recording of different durations during variable activities (*e.g.*, postural testing, physical or mental exercise, and controlled breathing) and at various stages of disease severity.

### 1.4 Recording Duration

Generally, long-term (*e.g.*, nominal 24 hours), short-term (5 minutes), and ultra-short-term (substantially shorter than 5 minutes) recordings have been used in HRV studies.

Long-term HRV analysis often utilizes 24-hour Holter monitoring or, less frequently, is based on data provided by simpler wearable devices. Indeed, such a long-term analysis allows circadian rhythms and daily variations to be captured in response to different activities and stressors. In addition to pathological confounding variables, the interpretation of long-term HRV analysis requires the consideration of many factors, including individual characteristics, nutrition, and psychosocial aspects. HRV may also be affected by common factors such as activity, mood, and stress. Therefore, it has been proposed that comparisons of long-term HRV measurements are valid only if all patients are subject to a standardized environment (*e.g.*, hospitalization) during long-term monitoring.

Short-term HRV assessments are based on electrocardiogram (ECG) recordings of 5 minutes, typically during rest or during the previously mentioned controlled conditions, such as exercise, controlled breathing, and postural testing. These can provide insights into the ANS responsiveness to provocative conditions [41]. ECG recordings of the standard 5-minute durations are commonly used for HRV analysis for several reasons: these recordings are relatively easy to obtain, it is possible to eliminate external influences during short-term recordings, and the standardized duration of the recordings allows valid comparisons to be performed [7, 15]. Importantly, controlled conditions during data acquisition allow HRV assessment in ambulatory patients [42].

Ultra-short-term (*i.e.*, much shorter than 5-minute, *e.g.*, 10-second) ECGs are standardly used for morphology/rhythm analysis but are problematic as a recommendation for HRV assessments [43, 44].

### 1.5 Recording Technology and Requirements

Single- or multiple-lead ECG is the most commonly used method for HRV data acquisition; however, plethysmography has also been used in certain HRV studies [45, 46]. In some plethysmography studies with healthy subjects, reasonable correlations were found between plethysmography-based and ECG-based HRV results [45, 46]. Thus, both methods are suitable for assessing heartbeat fluctuations. Because of sensor simplicity, plethysmography is frequently used in simpler commercial heart rate monitors, including those aimed at the home-based wearable device markets. However, plethysmography should not be considered a valid ECG surrogate (not only in HRV analyses) since ectopic activity and arrhythmias cannot be appropriately detected and classified. Additionally, increased arterial stiffness in older patients slows the pulse wave transit, further complicating HRV analysis. Intracardiac electrograms recorded by implantable devices are another possible data source for HRV analyses. However, the absence of valid NN interval sequences (*e.g.*, in patients with atrial fibrillation, frequent ectopy, or paced rhythms) makes it impossible to obtain valid HRV measurements.

## 2. HRV Measurements

Many different HRV indices have been proposed previously [15, 18]. In CHF studies, HRV has most frequently been expressed using either time-domain or frequency-domain quantifications. Time-domain measures express the extent of NN interval variability, and frequency-domain measures estimate the distribution of the absolute or relative power of NN variability in separate frequency bands [7, 15, 45, 47]. The interpretation of other HRV parameters is complex and, in some situations, controversial [18, 48].

### 2.1 Time-domain Methods

The time-domain indices represent perhaps the simplest HRV methods [15]. Usually, a continuous ECG signal is used, and durations of individual consecutive NN intervals are measured. Simple statistical descriptors may subsequently be derived from the NN interval sequence; the most frequently used are listed in Table 1. The mean NN interval or the mean heart rate is usually also derived. Additionally, simpler basic descriptors might be obtained, including the longest and shortest NN interval, their difference, and the day–night heart rate difference.

**Table 1.** **Selected HRV time-domain measures**.

Parameter	Description	Unit
SDNN	Standard deviation of all NN intervals	ms
SDANN	Standard deviation of NN averages in consecutive 5-minute segments	ms
RMSSD	Root mean square of successive differences between adjacent NN intervals	ms
NN50	Count of pairs of adjacent NN intervals that differ by more than 50 ms	-
pNN50	Count of pairs of adjacent NN intervals that differ by more than 50 ms divided by total number of NN intervals	%

HRV, heart rate variability; NN interval, normal-to-normal RR interval (the time elapsed between two successive sinus rhythm R-waves).

The numerical values of time-domain HRV indices depend, among others, on the duration of the analyzed recording. HRV index comparisons are only valid if the same period of the source recordings is used; otherwise, misleading conclusions might be reached [15].

The geometric HRV methods are related to time-domain techniques and are based on a graphical representation of the duration of the heart period. The common geometric methods include the HRV triangular index and the triangular interpolation of the NN interval histogram [15]. While these methods offer insights into the overall HRV patterns and dynamics, they have mainly been used to process long-term ECG recordings in which the detection of individual QRS complexes was not made with the highest accuracy [49, 50]. In addition to studies of CHF populations [51], these methods have also been applied to the heart period intervals detected by implantable devices [52].

### 2.2 Spectral-domain Methods 

Power spectral HRV analysis is usually based on standard technologies estimating signal spectral components, such as the Fast Fourier transform or parametric autoregressive modeling [15]. However, other possibilities have also been reported [46]. Four spectral components might be distinguished in the spectrum calculated from long-term recordings (*e.g.*, of nominal 24 hours): the ultra-low-frequency (ULF) <0.003 Hz, the very-low-frequency (VLF) 0.003–0.04 Hz, the low-frequency (LF) 0.04–0.15 Hz, and the high-frequency (HF) 0.15–0.4 Hz components [15, 40]. Only VLF (<0.04 Hz), LF, and HF components are used in short-term spectral analyses based on 5-minute data since it is not possible to obtain ULF measurements from 5-minute recordings. The physiological differences between the spectral components in HRV reflect the speed with which the ANS limbs and other heart rate-controlling mechanisms react to intrinsic demands and environmental challenges [53].

The HF component corresponds to parasympathetic heart rate modulations, mostly reflecting respiration-mediated heart rate changes [54, 55]. A correlation exists between HF and the time-domain measures manifesting parasympathetic modulations (pNN50 (count of pairs of adjacent NN intervals that differ by more than 50 ms divided by total number of NN intervals) and RMSSD (root mean square of successive differences between adjacent NN intervals)) [15, 54]. In healthy subjects, HF increases were observed under vagal dominance conditions [15, 29, 30].

The LF component is a mixture of parasympathetic and sympathetic modulations and is influenced by baroreceptor responses to blood pressure fluctuations [15, 56]. Compared to HF, LF reflects a more complex and not easily discernible mix of sympathovagal balance, renin–angiotensin activity, and baroreceptor reflexes [49, 56, 57]. Correlations were observed between LF power and time-domain SDNN (standard deviation of all NN intervals) and SDANN (standard deviation of NN averages in consecutive 5-minute segments) measures [15]. Furthermore, reported studies failed to correlate direct recording of sympathetic nerve activity with LF power, both in healthy subjects and in CHF patients [58, 59]. This highlights the fundamental difference between the sympathetic tone level and the extent of the sympathetic tone modulations [55].

The VLF component is influenced by many physiological mechanisms, *e.g.*, vasomotor tone, renin–angiotensin system, and thermoregulation [60]; however, true physiological correlates are yet to be established for VLF. Moreover, no physiological correlate exists for the ULF component. The ULF component was previously proposed for pure technical correspondence to the time-domain SDANN method (*i.e.*, aiming to exclude variability faster than a 5-minute cycle length). The influence of sleep patterns and consequential day–night differences in the heart rate have been proposed as possible contributors to the ULF components.

The ratio between LF and HF (LF/HF) has repeatedly been used to indicate the balance between sympathetic and vagal control [9]; thus, LF/HF is interpreted as an index of the sympathovagal balance [15, 61, 62]. Nevertheless, since some uncertainty remains regarding the physiological origins of the LF component, the physiological basis for LF/HF is difficult to discern with certainty, especially when considering conditions that affect the control mechanisms of sympathovagal balance [57, 63]. Moreover, if the HF component is very depressed, the statistical properties of the LF/HF ratio become problematic. Thus, interpreting the LF/HF ratio must consider individual characteristics and external influences to provide meaningful insight into cardiovascular health and autonomic regulation.

Normalized LF and HF (nLF and nHF) components, assessed standardly using 5-minute recordings, do not suffer from this problem [15, 56]. Indeed, nLF and nHF represent the relative value of each power component in proportion to the total power minus the VLF component. Fast Fourier transform spectral analysis allows only an approximate calculation of nLF and nHF as the true definition of nLF and nHF requires autoregressive spectral assessment, leading to additional components in the <0.4 Hz range.

Standard spectral HRV parameters are listed in Table 2 [15].

**Table 2.** **Standard frequency-domain HRV measures**.

Parameter	Description	Unit
Total spectral power	Frequency power 0–0.4 Hz	ms^2^
ULF	Ultra-low frequency power <0.003 Hz	ms^2^
VLF	Very low frequency power 0.003–0.04 Hz	ms^2^
LF	Low frequency power 0.04–0.15 Hz	ms^2^
HF	High frequency power 0.15–0.4 Hz	ms^2^
LF/HF	Ratio between the LF and HF components	-
nHF	Normalized HF component	-
nLF	Normalized LF component	-

According to the established standards [15], frequency-domain methods should be preferred to time-domain methods when investigating short-term recordings obtained under predetermined and consistent conditions. Indeed, spectral analysis over the entire 24-hour period obscures detailed information about autonomic modulation of NN intervals available in shorter recordings [64]. Thus, 5-minute recordings in a stationary condition are preferred unless the nature of the study dictates another design [15]. Sufficient sampling frequency and amplitude resolution of ECG signals are necessary to determine HRV parameters correctly. A low sampling frequency may cause digitization noise and errors in NN measurements. Meanwhile, several studies have suggested that a sampling frequency of 200 Hz might be sufficient [65]. However, a higher sampling frequency is preferred if not required in CHF because of the very low RR interval (the time elapsed between two successive sinus rhythm R-waves) variability and QRS pattern instability [7].

### 2.3 Nonlinear HRV Analysis

Nonlinear HRV analyses involve applications of mathematical and computational techniques of nonlinear dynamics and chaos theory. Unlike linear methods that assume constant relationships between variables, a nonlinear analysis acknowledges the intricate, dynamic, and often irregular nature of physiological systems [66]. The most common nonlinear HRV measurements include numerical processing of RR interval Lorenz plots [67], fractal analysis [68, 69], sample entropy [70], detrended fluctuation analysis [68, 70], and symbolic analyses [71]. Whilst these methods offer valuable technical insights, they also come with substantial limitations, including computational complexity and, more importantly, difficult physiologic interpretation and clinical evaluations that are not necessarily reproducible. Furthermore, nonlinear methods are difficult to standardize because they are sensitive to recording length, sampling rate, preprocessing techniques, and many other factors. Thus, comparing results across different studies or clinical settings is challenging [7, 17].

## 3. Review Methods

### 3.1 Systematic Literature Review

A systematic literature review was performed to assess CHF-induced ANS modifications, summarize autonomic HRV-based observations in CHF patients, and analyze reports of HRV usefulness in CHF risk stratification to review the applicability of HRV to CHF.

A PubMed literature search was performed from the database inception until June 2024 using the following search keywords: (“heart rate variability” OR “HRV”) AND (“heart failure”) AND (“prognosis” OR “all-cause mortality” OR “cardiac event” OR “sudden cardiac death”).

The following search results were obtained:

■ (“heart rate variability” OR “HRV”) AND (“heart failure”) AND (“prognosis”) — 240 results.

■ (“heart rate variability” OR “HRV”) AND (“heart failure”) AND (“all-cause mortality”) — 34 results.

■ (“heart rate variability” OR “HRV”) AND (“heart failure”) AND (“cardiac event”) — 7 results.

■ (“heart rate variability” OR “HRV”) AND (“heart failure”) AND (“sudden cardiac death”) — 111 results.

This initial search identified 392 publications. Duplicates, foreign language articles, and search results containing only an abstract without a full text were removed. From the remaining studies, we selected those that reported one or more of the following HRV parameters together with their numerical values:

■ Time-domain measures: SDNN, SDANN, RMSSD, pNN50.

■ Frequency-domain measures: LF, HF, LF/HF, nLF, nHF.

A total of 70 full-text articles remained for the detailed review.

### 3.2 Presentation of Published HRV Data

Numerical values of individual HRV measures were graphically displayed and used to describe the predictive values of individual HRV indices in CHF patients.

To study the overlap between HRV indices reported in CHF patients and healthy subjects, published studies were used if they reported the same HRV measurements and included numerical values of the mean and standard deviation of the populations. A case-by-case representation of the published population was modeled for each representative analysis using values taken randomly from the normal distribution with the detailed mean and standard deviation. For each analysis, the reported number of subjects was used as the number of repetitions of the randomly generated case-by-case modeling values. Subsequently, these case-by-case models of individual reported CHF populations were pooled to obtain the overall mean and standard deviation of the published HRV index in CHF patients. Subsequently, the same process was repeated to determine the overall mean and standard deviation of the published corresponding HRV values in healthy subjects. The modeled spans of HRV values in CHF patients and healthy subjects were used to display a general overlap between these clinical categories. This modeling approach was used when sufficient studies reporting the same HRV measurement were available.

## 4. Time-domain HRV Measurements in CHF Patients

Generally, time-domain HRV indices are accepted to decrease in CHF patients compared to healthy controls [34, 69, 70, 72].

### 4.1 Ultra-short-term Measurements

In healthy subjects, a good correlation (r > 0.9) between short-term (5-minute) and ultra-short-term (10-second to 1-minute) recordings was found only for the average RR duration and RMSSD [44]. Shaffer *et al*. [73] recorded 5-minute ECGs at rest and compared the resulting HRV parameters with those derived from shorter recordings (10 to 240 seconds), aiming at correlation coefficients of r ≥0.90. Sixty seconds was the minimum interval required for reasonable SDNN, RMSSD, NN50 (count of pairs of adjacent NN intervals that differ by more than 50 ms), and pNN50 correlations. Nevertheless, it remains questionable whether these results from a small study (only 38 healthy undergraduates) apply to other clinical populations, and particularly to CHF patients. Since recordings shorter than 2 minutes cannot properly capture sympathetic modulations [15], the usefulness of the ultra-short-term HRV measurements seems debatable, if not dubious.

### 4.2 Short-term Measurements

Only a few studies used short-term ECG recordings for the time-domain HRV analysis in CHF patients. The primary problem with reviewing the HRV results of short-term recordings is the variable recording length used in different studies. Significantly lower SDNN from short-term recordings was found during 15-minute rest, controlled breathing [74], and in 4-hour daytime segments in CHF patients [29] compared to non-VHF controls. However, only a few studies have analyzed HRV in CHF patients using exactly 5-minute recordings [37, 75, 76]. Meanwhile, none of these studies compared the HRV values in CHF patients with those in healthy subjects.

### 4.3 Long-term Measurements

SDNN is the most often used HRV index derived from long-term recordings in CHF patients. As already stated, the SDNN index is substantially influenced by the recording duration. Therefore, only results based on recordings of the same duration might be meaningfully compared. In nominal 24-hour ECG recordings, SDNN was generally reduced in CHF patients compared to healthy controls [32, 33, 77]. However, Guzzetti *et al*. [78] identified a non-significant trend toward reduced SDNN in CHF patients compared to healthy controls.

SDANN quantifies slow NN interval oscillations, mainly concentrating on diurnal variability [15, 79]. Thus, SDANN represents a suitable method for assessing day–night differences in heart rate oscillations. Plausibly, it may be expected that these differences are reduced in CHF patients. In recent studies, SDANN was calculated from continuous heart rate recordings by implantable devices to predict the outcome in CHF patients [80, 81, 82]. Patients who exhibited higher SDANN early after the implantation of cardiac resynchronization therapy (CRT) devices benefited more from the treatment.

Although it is generally accepted that under normal circumstances, both RMSSD and pNN50 predominantly measure oscillations in parasympathetic activity [16, 61], a study comparing CHF patients and healthy controls found no differences in RMSSD [32]. Further, RMSSD was not investigated in other studies, while pNN50 was significantly lower in CHF patients [29, 77]. Thus, CHF-related results of these HRV indices appear rather inconsistent.

**Fig. 1.**
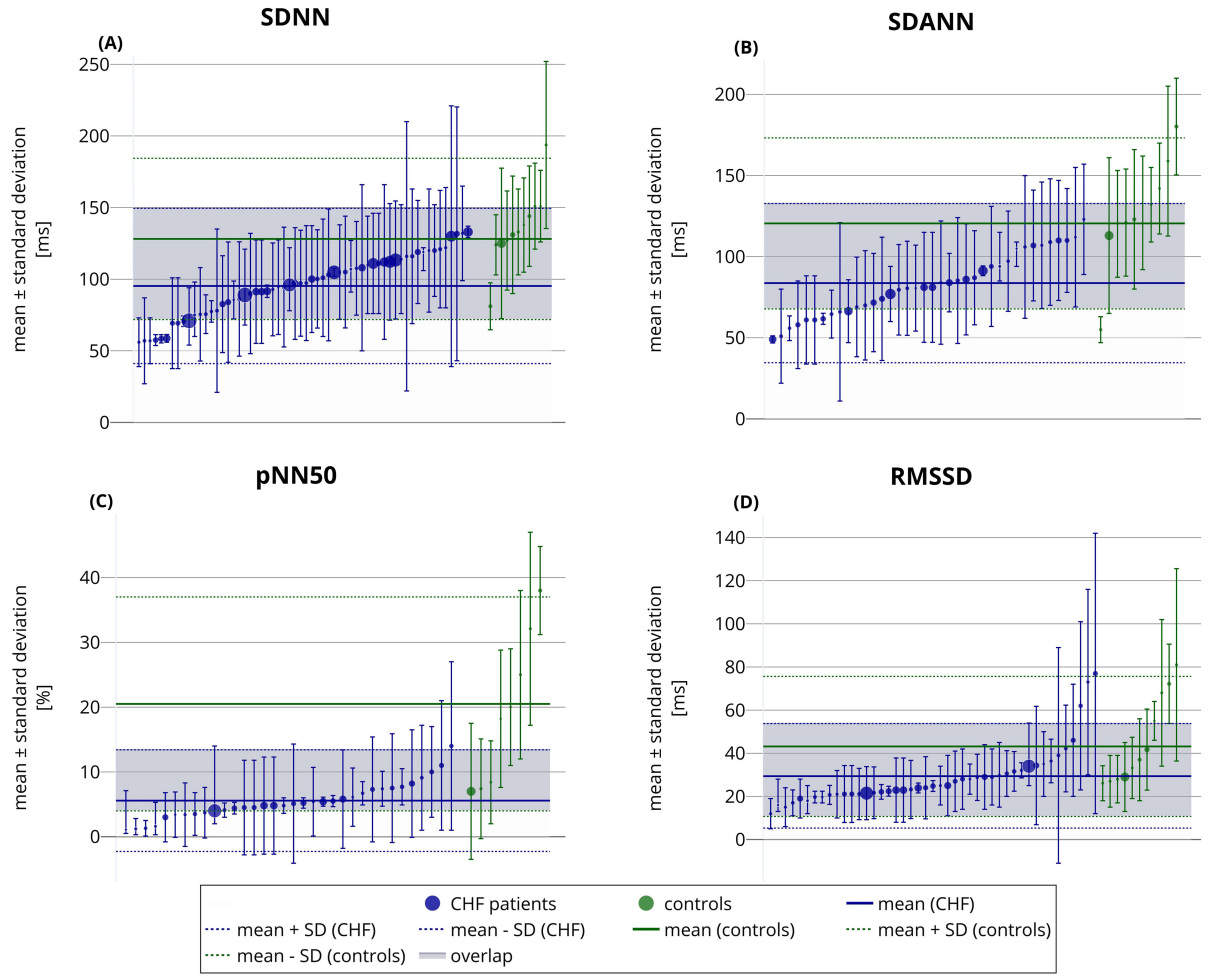
**Reported SDNN (A), SDANN (B), pNN50 (C), and RMSSD (D) values in reviewed studies of CHF patients (blue) and of healthy subjects (green)**. Each circle with error bars corresponds to a different publication. The circles present reported mean values, while the error bars show reported standard deviations. The circle sizes indicate the sample sizes used in the studies. The horizontal axis represents no variable and is used only for presentation purposes. The general modeled mean ± standard deviation bands are also shown for CHF patients and healthy subjects. The grey area shows the overlap between these bands (*i.e.*, the modeled overlap between published values in CHF patients and healthy subjects). All HRV values were obtained from long-term ECG recordings (nominal 24-hour Holter recordings). CHF, congestive heart failure; SD, standard deviation; ECG, electrocardiogram.

The tendency towards a decline in HRV time-domain measures in CHF patients is shown in Fig. 1. Nevertheless, an obvious overlap between the reported HRV numerical values in CHF patients and healthy controls is also evident (Fig. 1). Likewise, a wide variation in the reported HRV indices is visible within both clinical categories.

## 5. Frequency-domain HRV Measures in CHF Patients

Compared to the time-domain measures, the frequency-domain HRV indices are more sensitive to the accuracy of cardiac tachograms and may easily be influenced by artifacts due to heart rate instability in the analyzed recordings. This has evident implications for interpreting CHF studies using spectral HRV analysis.

A significant decrease in the HF component after a postural change from supine to standing and increased HF during controlled breathing was observed in healthy subjects [82]. In contrast, CHF patients failed to show any changes in the HF frequency induced by controlled breathing, tilt test, or postural changes [74, 82, 83]. However, decreasing respiration frequency from 20 to 10 breaths per minute led to a significant HF increase in CHF patients [82]. Nonetheless, the results of studies dealing with the HRV analysis during controlled breathing must be carefully interpreted. The HF modulations are linked to respiration, which influences short-term alterations in sinus rhythm frequency. Thus, changes in the HF component reflect differences in rhythm modulations that do not necessarily imply corresponding changes in the vagal tone [55].

Soejima *et al*. [54] observed HF decreases during early CHF stages but did not find any further progressive HF changes, in contrast to progressive LF decline. Similarly, Bonaduce *et al*. [84] concluded that the decrease in HF seems to occur early in CHF development; thus, HF is not a suitable marker to characterize CHF progression.

Previous studies indicated that the LF component is reduced by controlled respiration in normal subjects [74, 83]. During a postural test (position changes from supine to standing), the LF component increased in normal subjects regarding the total heart period variance [82]. In contrast, no significant changes in the LF component were observed in CHF patients after a postural change [82], tilt [83], or during controlled respiration [83]. Even in asymptomatic CHF patients, there was no difference in the LF component in response to these provocations. This might suggest that autonomic dysfunction occurs during early CHF stages [59, 74].

In a study using postural changes from supine to standing, an increase in the LF/HF ratio was seen in normal subjects; meanwhile, this was absent in CHF patients, possibly reflecting blunted cardiovascular reflexes [82].

An increased nLF was observed in healthy subjects during maneuvers that increase sympathetic tone (90° tilt, standing, mental stress, and moderate exercise) [85]. However, in the same study, CHF patients failed to show any nLF or nHF changes during the corresponding maneuvers. Guzzetti *et al*. [78] noted a significantly lower nLF spectral component in CHF patients than in healthy subjects.

Research using normalized spectral components remains limited. Some authors use a different frequency band for the LF spectrum (*e.g.*, 0.03–0.15 Hz) [78]. Meanwhile, because of the complex interpretation of the LF components, changes in LF/HF and normalized frequency measures are difficult to associate with specific autonomic control processes in CHF patients.

VLF and ULF were reported to have the strongest predictive value in acute myocardial infarction survivors [86]. Nevertheless, the predictive values of VLF and ULF in CHF patients remain uncertain. While some studies suggest a potential association between VLF components and cardiac events, only a few studies have investigated this relationship, and their findings are not easily comparable [87, 88, 89].

As discussed later in this text, numerical comparisons of spectral HRV results are problematic because of differences in reported units. Therefore, we did not attempt to apply the analyses presented in Fig. 1 (for the time-domain HRV indices) to the results of spectral HRV measurements.

## 6. CHF Prognosis and Progression

Risk stratification in CHF patients is clinically important. Patients at higher risk of adverse outcomes, such as hospitalization, worsening symptoms, or mortality, benefit from frequent clinical attention. Hence, effective risk stratification enables clinicians to tailor treatments with the aim of prognosis improvement [90]. Many published studies focused on all-cause mortality (ACM) and sudden cardiac death (SCD) in CHF patients. Conversely, numerous other studies have examined HRV predicting different cardiac events in CHF patients.

### 6.1 All-cause Mortality

**Fig. 2.**
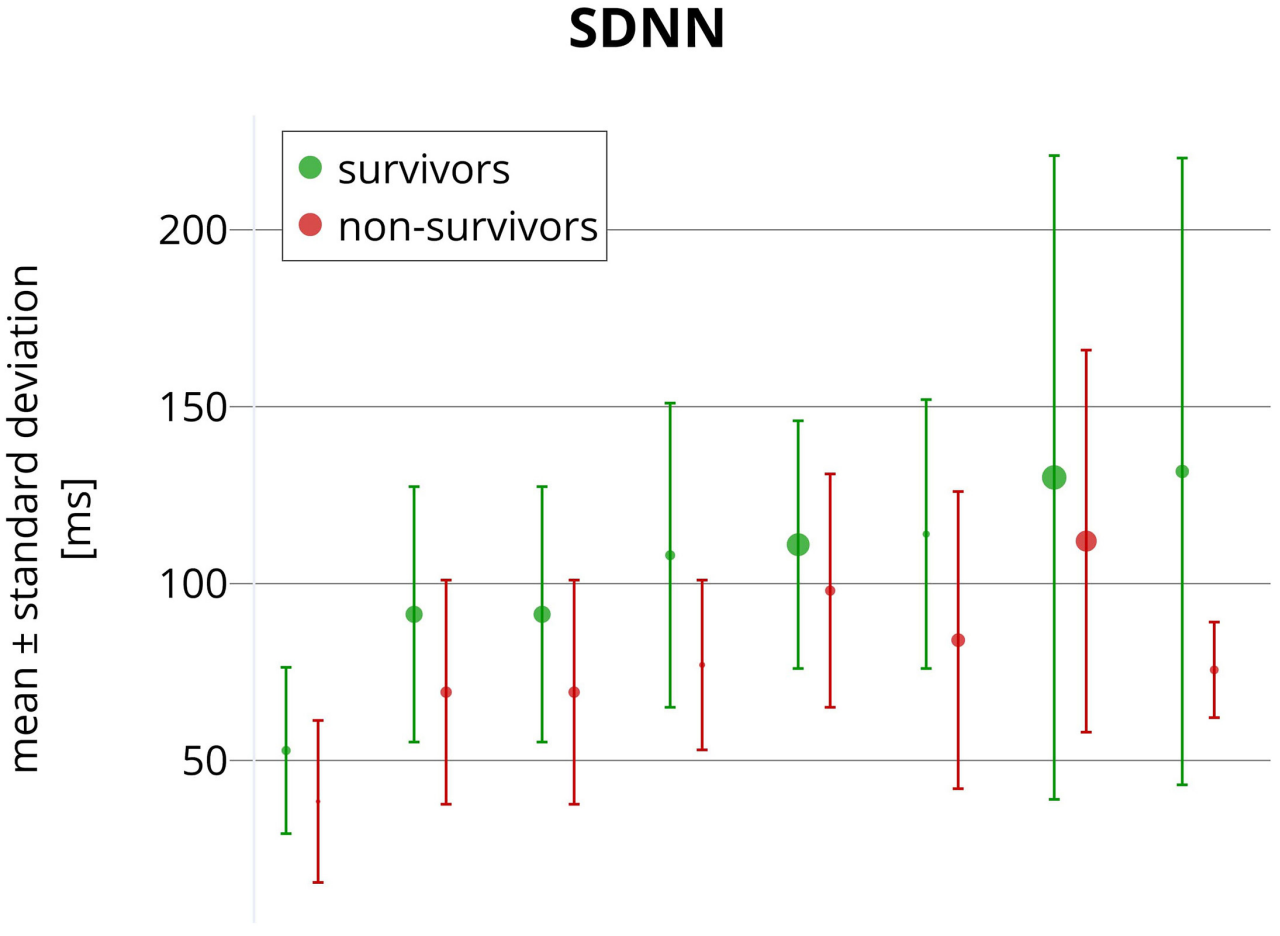
**Reported SDNN values comparing CHF survivors (green) and non-survivors (red)**. Each pair of circles with error bars corresponds to a different publication. Circles present mean values, while the error bars show reported standard deviations. The circle sizes indicate the sample sizes used in the studies. The horizontal axis represents no variable and is used only for presentation purposes. All SDNN values were obtained from long-term ECG recordings (nominal 24-hour Holter recordings).

It has long been debated whether decreased HRV indices indicate high ACM risk in CHF [16, 36]. In several studies, reduced SDNN values were associated with ACM in CHF patients [91, 92, 93]. However, different predictive values of the SDNN cut-off were proposed. Namely, 24-hour SDNN measurements below 108 ms [94], 100 ms [95], 86 ms [96], 70 ms [97], 67 ms [92], and 44 ms [39] were reported as ACM predictors in CHF patients. Meanwhile, among other studies, Nolan *et al*. [98] reported mortality rates in patients with mild to moderate CHF stratified according to SDNN measured over a nominal 24 hours and found ACM incidences of 5.5%, 12.7%, and 51.4% for SDNN measurements above 100 ms, between 50 and 100 ms, and below 50 ms, respectively. In a recent study by Cheng *et al*. [81], post-implant depressed HRV was associated with ACM in CHF patients receiving an implanted defibrillator with cardiac resynchronization function (CRT-D). For every 10 ms increase in SDNN at 6 months after CRT-D implantation, the risk of ACM decreased by 27%. Studies comparing 24-hour SDNN in CHF survivors and non-survivors are summarized in Fig. 2.

Using frequency-domain HRV analysis in CHF patients, baseline LF power has often been examined as an ACM predictor [93]. Ponikowski *et al*. [95] and Folino *et al*. [93] showed that reduced LF power identifies CHF patients at a high risk of cardiac death. In most studies, RMSSD, pNN50, and HF have not been found to predict ACM in CHF patients [93, 95, 98]. Further, the role of pNN50 in CHF risk stratification has been suggested only once [84].

Thus, compared to other HRV measures, SDNN and LF seem to be important prognostic ACM markers in CHF patients [92]. However, the published studies do not agree on a threshold that optimally indicates an increased ACM risk. Fig. 2 shows a large overlap of 24-hour SDNN values between surviving and non-surviving CHF patients.

### 6.2 Sudden Cardiac Death

SCD predictions based on HRV indices remain controversial in CHF patients [36]. Depressed SDNN values did not predict SCD in CHF patients with left ventricular ejection fraction (LVEF) greater than 35% [96]. The same observation was made in patients with mild to moderate CHF [91, 98, 99, 100]. In several studies, abnormal frequency components of HRV were also not found to predict SCD in mild to moderate CHF [54, 100].

Only a small number of studies showed significant differences in selected HRV measures between SCD victims and survivors among CHF patients. Galinier *et al*. [92] found that reduced daytime LF power was an independent risk predictor of SCD in CHF patients with a New York Heart Association (NYHA) class above stage I. In the same study, SDANN and RMSSD were significantly related to SCD. Fauchier *et al*. [101] reported that depressed SDNN (<100 ms) was an independent risk predictor of SCD and arrhythmic events in patients with idiopathic dilated cardiomyopathy. La Rovere *et al*. [37] showed that reduced short-term LF during controlled respiration was an independent SCD predictor in CHF patients.

Limited numbers of SCD events during the follow-up periods of reported studies are likely one of the reasons for the observed inconsistencies. Additionally, many CHF patient deaths are difficult to classify with certainty [92, 96, 102]. Multiple studies also suggested that in CHF patients with lowered LVEF, ischemic heart disease and/or non-sustained ventricular tachycardias are more potent SCD predictors than HRV indices [92, 98, 99, 100]. Many other studies on CHF patients also reported that the risk was not purely associated with SCD but the risk of SCD in combination with other cardiac events [32, 42, 87].

### 6.3 Other Cardiac Events

Similar to SCD risk stratification, HRV-based predictions of combined cardiac events (CEs) in CHF patients also appear controversial. Studies have been reported that include a wide variety of end-point classifications: The combination of SCD, pump failure death, transplantation, and hospitalization due to CHF worsening has frequently been used [32, 42, 87, 88, 89, 103, 104]. Kaufmann *et al*. [105] used a major adverse cardiovascular events endpoint, defined as a combination of cardiovascular death, nonfatal myocardial infarction, and/or nonfatal stroke, while using short 10-minute recordings during controlled respiration in ischemic CHF patients. Furthermore, Kaufmann *et al*. [105] reported that none of the HRV indices were significant CE predictors.

Likewise, the reported CE risk association with the LF component and the LF/HF ratio appears inconsistent. While the LF/HF ratio did not predict CE in most studies [87, 88, 105, 106], Lucreziotti *et al*. [42] described a low LF/HF ratio as an independent CE predictor in CHF. A possible explanation may be including patients with advanced disease stages; thus, spectral analysis might allow better prognostic stratification in advanced CHF stages [54].

Conversely, SDNN appeared to be a promising CE predictor [32, 95, 103, 104, 107]. Jiang *et al*. [104] observed that depressed SDNN had a higher predictive value than more conventional parameters (lower LVEF and increased frequency of ventricular premature complexes) that were not associated with CE. Nevertheless, other studies did not demonstrate the predictive value of SDNN [88, 105, 106]. Krüger *et al*. [103] concluded that in CHF patients, the inclusion of depressed SDNN enhances the prognostic power offered by lowered LVEF. However, other HRV indices failed to predict CE in this study [103].

RMSSD and pNN50 did not show any usefulness in CE risk stratification studies. This finding is unsurprising since these indices express rapid beat-to-beat heart period changes related to abnormal regulatory systems in most CHF patients [98]. Even more controversial is the predictive power of frequency-domain HRV indices [36]. Thus, no specific link has been established between CE and parasympathetic and/or sympathetic activity and sympathovagal balance [93].

Studies using HRV measures for predicting various CE measures are shown in Table 3 (Ref. [32, 42, 87, 88, 89, 95, 103, 104, 105, 106, 107]). Table 3 illustrates the difficulty in comparing the studies since different CE definitions prevent overall conclusions from being made.

**Table 3.** **The prognostic significance of HRV measures in predicting CE in CHF patients**.

Study	Recording	Patient numbers	Cardiac events	Follow-up	Findings
Fauchier *et al*., 1997 [32]	24-hour Holter	93 IDC pts: 33 NYHA I and 60 NYHA II–IV	SCD, pump failure death, and transplantation	49.5 ± 35.6 months	Reduced SDNN independent predictor of CE; RMSSD not predictive of CE
Krüger *et al*., 2002 [103]	24-hour Holter	222 CHF pts	SCD, pump failure death, and CHF hospitalization	15 ± 1 months	SDNN was significantly lower in patients with CE
Fauchier *et al*., 2003 [107]	24-hour Holter	103 IDC pts	Cardiac related death, sustained ventricular tachycardia, and transplantation	27 ± 23 months	SDNN was an independent predictor of CE
Jiang *et al*., 1997 [104]	24-hour Holter	26 CHF pts of NYHA III	SCD, pump failure death, and successful resuscitation	18 months	SDNN and SDANN are significantly lower in patients with CE.
					RMSSD, pNN50, LF, and HF are not predictive of CE
Ponikowski *et al*., 1997 [95]	24-hour Holter	102 CHF pts:	Cardiac-related death	584 ± 405 days	SDNN, SDANN, and LF are independent predictors of survivors.
		48 NYHA II, 51 NYHA III, and 3 NYHA IV			
					RMSSD, pNN50, and HF are not predictive of CE
Lucreziotti *et al*., 2000 [42]	5 minutes	75 CHF pts	SCD, pump failure death, and transplantation	11.4 (0.20–38.40) months	LF/HF is an independent predictor of CE.
					LF and HF are not predictive for CE
Tateishi *et al*., 2002 [89]	24-hour Holter	43 CHF pts:	SCD and CHF hospitalization	12 months	VLF can be used in Cheyne–Stokes breathing patients to predict CE
		33 NYHA II and 11 NYHA III			
Yamada *et al*., 2003 [88]	24-hour Holter	65 CHF pts	SCD and CHF hospitalization	34 ± 19 months	VLF is significantly associated with CE.
					SDNN, SDANN, RMSSD, pNN50, LF, HF, and LF/HF are not predictive of CE
Hadase *et al*., 2004 [87]	24-hour Holter	54 CHF pts	SCD, pump failure death, and CHF hospitalization	19.8 ± 11.7 months	VLF is an independent predictor of CE.
					HF and LF/HF are not predictive of CE
Hashimoto *et al*., 2020 [106]	24-hour Holter	133 CHF pts	Cardiac related death, acute myocardial infarction, stroke, and CHF hospitalization	5.4 ± 4.1 years	VLF (night-time) is significantly lower in patients with CE.
					SDNN and LF/HF (daytime, nighttime, total) are not predictive of CE
Kaufmann *et al*., 2022 [105]	10 minutes	188 ich CHF pts:	Cardiovascular death, nonfatal myocardial infarction, and nonfatal stroke	34 (14–71) months	SDNN, RMSSD, pNN50, nLF, and LF/HF are not predictive of CE
		34 NYHA I, 122 NYHA II, and 32 NYHA III			

CE, cardiac event; ich, ischemic; IDC, idiopathic-dilated cardiomyopathy; LF/HF, the ratio between the low-frequency and high-frequency; NYHA, New York Heart Association; pts, patients; SCD, sudden cardiac death.

## 7. Discussion

The published literature shows clear agreements, substantial disagreements, and existing open questions related to recording technology, HRV measurements, and their clinical value and applicability.

### 7.1 Agreements

#### 7.1.1 Recording Technology

Ultra-short-term HRV analyses have not been found useful in CHF research. Meanwhile, only a few studies utilized ultra-short-term HRV analysis in CHF patients. Shah *et al*. [108] found that reduced baseline HRV (determined by SDNN and RMSSD) is associated with an increased incidence of CHF. Nevertheless, according to the established standards [15], the LF component cannot be estimated from recordings shorter than 2 minutes (for reliable spectra, stable 5-minute recordings are recommended). Thus, ultra-short ECG recordings are unsuitable for spectral HRV analysis and are not only for CHF patients [15].

Short-term HRV analyses are commonly used in CHF severity, progression, or mortality prediction studies. While the number of these studies is not large, short-term ECG recordings are easy to obtain, and their advantages include faster data processing and, more importantly, the convenience of controlling confounding factors (activity, respiration, body position, and room temperature). In contrast, short-term HRV analyses might suffer from lower reproducibility due to the physiologic variation in the cardiac autonomic status [12].

In comparison, long-term HRV analysis is a more stable tool, provided that standardized recording conditions are ensured. Consequently, most studies comparing HRV in CHF patients have been based on ECG signals of nominal 24-hour Holter recordings; even longer recordings might be obtained from wearable electronics or implantable devices. Nevertheless, much longer recordings are, by definition, more time-consuming to determine and might easily be influenced by changes in physical activity, environmental factors, and noise [12].

#### 7.1.2 HRV Measurements

Lower SDNN values are commonly observed in CHF patients, representing reduced autonomic flexibility due to impaired cardiac hemodynamic function. Numerous studies have demonstrated that lower SDNN values are associated with worse clinical outcomes, including higher mortality rates [32, 95, 103, 104, 107]. In CHF studies, SDNN and other HRV parameters were most frequently obtained from nominal 24-hour ECG recordings.

#### 7.1.3 Clinical Applicability

Preserved autonomic responsiveness characterizes CHF patients with better prognosis [93]. Simple maneuvers (*e.g.*, postural tests or controlled breathing) that allow autonomic responsiveness to be assessed based on short-term HRV comparisons have been used in several clinical outcomes studies in CHF patients [37]. Physical activity, posture, and respiratory effort influence HRV, yet these confounders are all difficult to standardize during long-term ambulatory recordings [109]. Thus, short-term ECG recordings obtained under the supervision of the investigator are recommended. Short-term data acquisition during provocative maneuvers seems to provide more useful information regarding CHF prognosis than evaluating a baseline unprovoked cardiac autonomic status [93]. HRV assessed from these recordings appears useful in identifying CHF patients likely to benefit from CRT [110, 111] and might also identify patients with better prognoses [93]. CHF patients with poor prognosis show more rigid autonomic responses, or a lack of such responses, as they are unable to react to many provocative stimuli. Indeed, significant HRV variation is missing during simple, provocative maneuvers in CHF patients with the worst prognosis [93]. The reproducibility of all these observations depends, to a large extent, on the conditions and duration of ECG recordings, which, unfortunately, have not been consistent in published studies.

Several authors analyzed long-term HRV in CHF patients indicated for CRT implantation [80, 111, 112, 113]. Implanted CRT devices might be used to measure HRV and monitor HRV evolution continuously after implantation. Meanwhile, studies showed HRV improvement in CHF patients with left bundle block after CRT implantation [81, 110]. A lack of HRV improvement after CRT implantation also identifies patients at higher risk of CE, ventricular tachycardia, and/or ACM [81, 111, 113].

Clinical use of HRV is also limited for other reasons. The prevalence of atrial fibrillation, which eliminates the possibility of HRV assessment, is around 10–15% in mild to moderate CHF and increases up to 50% in patients with more advanced CHF [114]. HRV analysis may be negatively influenced by atrial and ventricular ectopic beats or atrial stimulated beats in CHF patients with cardiac implantable devices [115]. Even if the ectopic beats are removed from the recording, the effects of heart rate turbulence remain [116, 117] and tend to increase the LF power without affecting the HF power appreciably [115]. Sinus node dysfunction, atrioventricular block, and prevalent paced rhythm are additional exclusion criteria in HRV studies of CHF patients [88].

### 7.2 Disagreements

#### 7.2.1 Recording Technology

Diurnal physical activity, breathing, and cognitive and emotional reactions influence HRV while being difficult to standardize during long-term recordings [109, 118]. Additionally, published articles often do not describe the conditions under which the 24-hour Holter recordings were obtained.

#### 7.2.2 HRV Measurements

Although most studies have shown depressed HRV in CHF patients, a wide variation exists among the reported time-domain HRV indices. We have already shown an overlap in time-domain HRV measures between CHF patients and healthy subjects. Therefore, numerical differences in HRV indices between CHF patients and normal controls are obscured, likely because of dissimilarities in investigative protocols and recording conditions.

Spectral analysis of HRV benefits from standardized 5-minute ECG recordings. However, while such recordings are frequently used [37, 119], many studies used different setups, including 10-minute or longer recordings [119, 120]. Moreover, many authors used spectral analysis of 24-hour recordings or shorter segments (*e.g.*, 5 minutes) with results averaged over the entire 24-hour period [31, 37, 42, 74, 82, 119, 121, 122, 123].

The reported units for the main spectral components (VLF, LF, HF) are also not uniform. In most studies, the appropriate units of *ms*^2^ were used. However, some authors reported units of *ln(ms^2^), ln(ms), (beats/min)^2^ × 10^-3^*, some of which are not necessarily easy to recalculate into the standard *ms*^2^ units. This makes comparison of spectral components in CHF patients difficult.

#### 7.2.3 Clinical Applicability

Although several studies confirmed that SDNN provides independent prognostic value in CHF patients [98, 124], no consistent SDNN cut-off values have been reported. Thus, while reported sensitivity might be high, lower specificity prevents any measure from being used alone as a routine screening test [125, 126]. The reported results regarding predicted SCD and/or other CE remain conflicting.

A general consensus has emerged that HRV indices decrease as the CHF severity increases. This is consistent with increased sympathetic activation due to disease progression [109]. However, the published studies characterizing CHF severity are less consistent. A significant reduction in SDNN, SDANN, and RMSSD was found in CHF patients with NYHA classes ≥ II compared to NYHA class I [127]. Nevertheless, differences in HRV among the NYHA classes II, III, and IV have not been systematically investigated. Yi *et al*. [33] suggested that 24-hour SDNN values below 50 ms predict the development of progressive heart failure (*i.e.*, NYHA class deterioration). However, another study noted that only a night-time analysis of SDNN and SDANN values detected differences between NYHA classes [128].

The use of spectral HRV analysis in assessing CHF severity remains even more controversial. A significant increase in the LF spectral component was found in mild symptomatic CHF patients (NYHA class II). This may reflect decreased sinus nodal responsiveness to neural modulatory influences [59, 83]. However, another study found no significant difference in LF power between patients with asymptomatic left ventricular dysfunction and normal subjects [74]. The LF component seems to decrease with the severity of the disease [74], and an almost complete absence of LF was found in the most severe CHF of NYHA class IV [79, 83]. A possible explanation for the reduction in LF might include impaired adrenergic receptor responsiveness, central autonomic regulatory impairment, and increased chemoreceptor sensitivity [30, 48]. Soejima *et al*. [54] showed LF decreases progressively depending on the severity of CHF, while the LF/HF ratio did not show any significant differences between NYHA classes. Nevertheless, other studies failed to document any significant correlation between declined spectral HRV indices and NYHA functional class [42, 128, 129].

### 7.3 Open Questions

HRV assessment is not commonly used in clinical practice for reasons that likely include insufficient validation, inconsistent results, and methodological complexity. Long-term HRV assessment ideally requires in-hospital recordings, so the healthcare burden becomes a practical obstacle [130, 131, 132]. Therefore, it is appropriate to consider key areas for future research to enhance the understanding of the remaining problems and to increase the applicability of HRV in CHF management.

#### 7.3.1 Recording Technology

Accurate HRV measurements require consistent, high-quality data, which may be challenging, especially with long-term recording, as artifacts and recording inconsistencies might pollute these data. Conversely, short-term measurements might eliminate these problems. However, the question remains of how to perform a short, standardized test that could be applied in a standard outpatient environment. The recording conditions (duration, posture, free or controlled breathing, mood, stress, *etc.*) can substantially affect the results of short-term HRV measures. Thus, broadly acceptable investigative protocols are needed to ensure standardized conditions during a short HRV test.

#### 7.3.2 HRV Measurements

Numerous HRV indices might be derived from ECG recordings. As discussed, a spectral analysis offers advantages over time-domain measures when applied to short-term recordings that would make HRV-based testing more practical. In conjunction with provocative components of the HRV tests, standardization of the technical set-up of the spectral HRV analysis is needed, including the selection of outcome parameters.

Nonlinear HRV methods were not included in our review due to their limited clinical applicability [18] and lack of sufficient validation in CHF studies, which often involved only small numbers of patients.

It might appear appealing to involve artificial intelligence (AI) systems in supporting future HRV analyses. Indeed, AI has already been successfully applied to risk assessment in well-defined clinical populations [133, 134, 135]. Nevertheless, it is not obvious whether and how AI might address the problems of handling recordings of incompatible quality and duration (which might, despite their poor reproducibility, be selected by the AI models as prognostic indicators instead of the HRV indices) and what input should be provided to the AI systems. Thus, if the source ECG recordings are processed, characteristics very different from the sinus nodal periodicity might dominate the AI systems [136, 137].

#### 7.3.3 Clinical Applicability

HRV analysis should be capable of enhancing the personalized healthcare of CHF patients by providing insights into autonomic function and facilitating continuous monitoring. ANS responds differently to various stimuli, situations, and treatments for each patient, which all necessitate a tailored approach. Thus, methodologies need to be proposed to establish a baseline HRV profile for each CHF patient and utilize such profiles to optimize therapeutic interventions.

HRV analysis might also be a cost-effective tool for monitoring and predicting responses to CHF treatment, including medication, CRT, and lifestyle modifications. However, different CRT device manufacturers implement different HRV measures, leading to inconsistencies in available parameters and making their global interpretation difficult [111, 138, 139]. This presently limits any standardized use of HRV in therapy assessment. An increase in HRV may indicate improved autonomic regulation and cardiac function, whereas a lack of improvement or a decline in HRV indices might suggest a suboptimal response to CRT. In such cases, HRV data could potentially guide CRT optimization by suggesting adjustments to device settings. Advanced methods, such as individually tailored AV and VV synchronization using ultra-high-frequency ECG, may further refine therapeutic outcomes.

Wearable devices have gained significant interest for their potential in monitoring CHF patients [140]. Indeed, most commercially available smartwatches rely on photoplethysmography to detect heart rate and cardiac cycle periods [141]. Nevertheless, as already mentioned, although convenient and noninvasive, photoplethysmography is susceptible to motion artifacts, changes in skin tone, and varying levels of ambient light, which all might compromise the accuracy of HRV measurements [142].

### 7.4 Recommendations for HRV Study Standards

Key standardization questions include (a) which HRV measure is the most reliable predictor of CRT response and (b) how HRV-based optimization strategies might be effectively implemented in clinical practice.

To ensure standardized conditions and minimize external stressors, HRV testing should occur in a noise- and disturbance-free environment, including temperature-controlled examination rooms. Moreover, a 5–10-minute rest before the test should be included to stabilize heart rate, and a standing, seated, or supine position is typically used, as postural changes affect autonomic tone. A combination of several postural positions maintained for 10 minutes (of which the middle 5-minute section is subsequently used for the HRV analysis) makes it possible to assess ANS behavior and responses to the postural provocations. This has already been shown in a large pediatric population of healthy children and adolescents [143, 144, 145]. Nevertheless, extensive protocols with many postural changes are likely unnecessary since the increased duration of the tests might hamper their clinical applicability. Ideally, tests should be concise to ensure feasibility in a clinical environment.

The selection of HRV indices and the definition of their cut-off thresholds are critical for the effectiveness and applicability of HRV analysis. As we have shown, the most useful HRV indices in CHF patients were obtained from long-term recordings; hence, choosing a suitable index for our proposed short tests might be challenging. Therefore, further experience with the proposed postural provocative tests is needed.

The time-domain and frequency-domain measures are widely accepted and should be considered. Consistent with the recent consensus on developing more advanced HRV techniques [18], further experience with the well-established time- and frequency-domain methods seems to be fundamentally preferable to creating newer, more complex, and technically challenging methods. More importantly, establishing well-defined cut-off thresholds for the time- and frequency-domain methods now represents an unmet clinical need, and solving it involves extensive research correlating specific HRV indices values with clinical outcomes in CHF patients. Additionally, combining HRV indices with the characterization of myocardial substrate might enhance clinical insight into CHF prognosis.

### 7.5 Limitations

While reviewing the literature on the diagnostic and prognostic roles of various HRV indices in CHF patients, we have intentionally limited the scope of the review to the time-domain and spectral-domain HRV techniques. More technically complex, especially non-linear, measures have not been considered since these do not appear to offer noticeable clinical advantages to the more established indices. Indeed, a PubMed search yielded only 34 nonlinear HRV studies, mostly with fewer than 50 patients. A detailed comparison of spectral components was also not included. The corresponding published articles used different recording durations and inconsistent reporting units—we could not reconcile these differences. Moreover, it would be valuable to divide the article by other types of heart failure (systolic/diastolic, heart failure with preserved/reduced/mildly reduced ejection fraction, *etc.*); however, very few articles deal with HRV in these CHF sub-populations. Consequently, a review of these might have been misleading. While focusing on HRV in CHF patients, we have not considered age, sex, or race when comparing individual HRV measures. The modeling approach to derive globally representative HRV data in healthy subjects and CHF patients should have ideally included Monte Carlo-type repetitions of random number generation. Nevertheless, since our aim was merely to demonstrate a substantial overlap between healthy subjects and CHF patients, we trust that our simpler approach was fully sufficient.

## 8. Conclusion

In summary, there is a general agreement that HRV is decreased in CHF patients. Despite expectations, simple comparisons of CHF patients with healthy subjects did not find any clear and fully consistent separation of the two population categories; meanwhile, using HRV indices as risk factors in CHF patients remains controversial. While time- and frequency-domain analyses were repeatedly reported to predict ACM, no apparent agreement exists on the cut-off values for any of these HRV indices. Thus, the HRV-based prediction of SCD and other CE in CHF patients does not appear clinically valuable.

Consequently, we propose several standardization questions and open issues concerning the established time- and spectral-domain HRV indices. Short-term recordings are preferred for practical reasons, as these allow a standardized environment and repeatable investigation protocol to be proposed and tested. We strongly believe that for the clinically practical utility of HRV assessment in CHF patients, solutions to these problems are needed much more than developing even more complicated HRV techniques, regardless of their conceptually or technologically elaborate concepts.

## References

[1] Lüscher TF (2015). Heart failure: the cardiovascular epidemic of the 21st century. *European Heart Journal*.

[2] Ponikowski P, Anker SD, AlHabib KF, Cowie MR, Force TL, Hu S (2014). Heart failure: preventing disease and death worldwide. *ESC Heart Failure*.

[3] Ponikowski P, Voors AA, Anker SD, Bueno H, Cleland JGF, Coats AJS (2016). 2016 ESC Guidelines for the diagnosis and treatment of acute and chronic heart failure. *European Heart Journal*.

[4] King M, Kingery J, Casey B (2012). Diagnosis and evaluation of heart failure. *American Family Physician*.

[5] Jong TL, Chang B, Kuo CD (2011). Optimal timing in screening patients with congestive heart failure and healthy subjects during circadian observation. *Annals of Biomedical Engineering*.

[6] Arsenos P, Gatzoulis KA, Dilaveris P, Sideris S, Tousoulis D (2018). T wave alternans extracted from 30-minute short resting Holter ECG recordings predicts mortality in heart failure. *Journal of Electrocardiology*.

[7] Shaffer F, Ginsberg JP (2017). An Overview of Heart Rate Variability Metrics and Norms. *Frontiers in Public Health*.

[8] Nunan D, Sandercock GRH, Brodie DA (2010). A quantitative systematic review of normal values for short-term heart rate variability in healthy adults. *Pacing and Clinical Electrophysiology: PACE*.

[9] Borovac JA, D’Amario D, Bozic J, Glavas D (2020). Sympathetic nervous system activation and heart failure: Current state of evidence and the pathophysiology in the light of novel biomarkers. *World Journal of Cardiology*.

[10] Nolan J, Flapan AD, Capewell S, MacDonald TM, Neilson JM, Ewing DJ (1992). Decreased cardiac parasympathetic activity in chronic heart failure and its relation to left ventricular function. *British Heart Journal*.

[11] Barthel P, Bauer A, Müller A, Huster KM, Kanters JK, Paruchuri V (2012). Spontaneous baroreflex sensitivity: prospective validation trial of a novel technique in survivors of acute myocardial infarction. *Heart Rhythm*.

[12] Li K, Rüdiger H, Ziemssen T (2019). Spectral Analysis of Heart Rate Variability: Time Window Matters. *Frontiers in Neurology*.

[13] Ziemssen T, Reimann M, Gasch J, Rüdiger H (2013). Trigonometric regressive spectral analysis: an innovative tool for evaluating the autonomic nervous system. *Journal of Neural Transmission (Vienna, Austria: 1996)*.

[14] Di Rienzo M, Parati G, Radaelli A, Castiglioni P (2009). Baroreflex contribution to blood pressure and heart rate oscillations: time scales, time-variant characteristics and nonlinearities. *Philosophical Transactions. Series A, Mathematical, Physical, and Engineering Sciences*.

[15] (1996). Heart rate variability: standards of measurement, physiological interpretation and clinical use. Task Force of the European Society of Cardiology and the North American Society of Pacing and Electrophysiology. *Circulation*.

[16] Sandercock GRH, Brodie DA (2006). The role of heart rate variability in prognosis for different modes of death in chronic heart failure. *Pacing and Clinical Electrophysiology: PACE*.

[17] Işler Y, Kuntalp M (2007). Combining classical HRV indices with wavelet entropy measures improves to performance in diagnosing congestive heart failure. *Computers in Biology and Medicine*.

[18] Sassi R, Cerutti S, Lombardi F, Malik M, Huikuri HV, Peng CK (2015). Advances in heart rate variability signal analysis: joint position statement by the e-Cardiology ESC Working Group and the European Heart Rhythm Association co-endorsed by the Asia Pacific Heart Rhythm Society. *Europace: European Pacing, Arrhythmias, and Cardiac Electrophysiology: Journal of the Working Groups on Cardiac Pacing, Arrhythmias, and Cardiac Cellular Electrophysiology of the European Society of Cardiology*.

[19] Mejía-Mejía E, Budidha K, Abay TY, May JM, Kyriacou PA (2020). Heart Rate Variability (HRV) and Pulse Rate Variability (PRV) for the Assessment of Autonomic Responses. *Frontiers in Physiology*.

[20] Ghuran AV, Malik M, Malik M (1998). Influence of smoking, alcohol, caffeine and recreational drugs on cardiac autonomic tests. *Clinical Guide to Cardiac Autonomic Tests*.

[21] Eckberg DL (1997). Sympathovagal balance: a critical appraisal. *Circulation*.

[22] Parati G, Saul JP, Di Rienzo M, Mancia G (1995). Spectral analysis of blood pressure and heart rate variability in evaluating cardiovascular regulation. A critical appraisal. *Hypertension (Dallas, Tex.: 1979)*.

[23] Barthel P, Bauer A, Müller A, Junk N, Huster KM, Ulm K (2011). Reflex and tonic autonomic markers for risk stratification in patients with type 2 diabetes surviving acute myocardial infarction. *Diabetes Care*.

[24] Hansen CS, Vistisen D, Jørgensen ME, Witte DR, Brunner EJ, Tabák AG (2017). Adiponectin, biomarkers of inflammation and changes in cardiac autonomic function: Whitehall II study. *Cardiovascular Diabetology*.

[25] Hansen CS, Færch K, Jørgensen ME, Malik M, Witte DR, Brunner EJ (2019). Heart Rate, Autonomic Function, and Future Changes in Glucose Metabolism in Individuals Without Diabetes: The Whitehall II Cohort Study. *Diabetes Care*.

[26] Chandola T, Britton A, Brunner E, Hemingway H, Malik M, Kumari M (2008). Work stress and coronary heart disease: what are the mechanisms?. *European Heart Journal*.

[27] Chatterjee A, Riegler MA, Ganesh K, Halvorsen P (2025). Stress management with HRV following AI, semantic ontology, genetic algorithm and tree explainer. *Scientific Reports*.

[28] Kleiger RE, Stein PK, Bigger JT (2005). Heart rate variability: measurement and clinical utility. *Annals of Noninvasive Electrocardiology: the Official Journal of the International Society for Holter and Noninvasive Electrocardiology, Inc.*.

[29] Tsai CH, Ma HP, Lin YT, Hung CS, Huang SH, Chuang BL (2020). Usefulness of heart rhythm complexity in heart failure detection and diagnosis. *Scientific Reports*.

[30] Guzzetti S, Magatelli R, Borroni E, Mezzetti S (2001). Heart rate variability in chronic heart failure. *Autonomic Neuroscience: Basic & Clinical*.

[31] Notarius CF, Floras JS (2012). Caffeine Enhances Heart Rate Variability in Middle-Aged Healthy, But Not Heart Failure Subjects. *Journal of Caffeine Research*.

[32] Fauchier L, Babuty D, Cosnay P, Autret ML, Fauchier JP (1997). Heart rate variability in idiopathic dilated cardiomyopathy: characteristics and prognostic value. *Journal of the American College of Cardiology*.

[33] Yi G, Goldman JH, Keeling PJ, Reardon M, McKenna WJ, Malik M (1997). Heart rate variability in idiopathic dilated cardiomyopathy: relation to disease severity and prognosis. *Heart (British Cardiac Society)*.

[34] Francis GS, Benedict C, Johnstone DE, Kirlin PC, Nicklas J, Liang CS (1990). Comparison of neuroendocrine activation in patients with left ventricular dysfunction with and without congestive heart failure. A substudy of the Studies of Left Ventricular Dysfunction (SOLVD). *Circulation*.

[35] Ferguson DW, Berg WJ, Roach PJ, Oren RM, Mark AL (1992). Effects of heart failure on baroreflex control of sympathetic neural activity. *The American Journal of Cardiology*.

[36] Cygankiewicz I, Zareba W, de Luna AB (2008). Prognostic value of Holter monitoring in congestive heart failure. *Cardiology Journal*.

[37] La Rovere MT, Pinna GD, Maestri R, Mortara A, Capomolla S, Febo O (2003). Short-term heart rate variability strongly predicts sudden cardiac death in chronic heart failure patients. *Circulation*.

[38] Cygankiewicz I, Zareba W, Vazquez R, Vallverdu M, Gonzalez-Juanatey JR, Valdes M (2008). Heart rate turbulence predicts all-cause mortality and sudden death in congestive heart failure patients. *Heart Rhythm*.

[39] Aronson D, Mittleman MA, Burger AJ (2004). Measures of heart period variability as predictors of mortality in hospitalized patients with decompensated congestive heart failure. *The American Journal of Cardiology*.

[40] Pecchia L, Melillo P, Bracale M (2011). Remote health monitoring of heart failure with data mining via CART method on HRV features. *IEEE Transactions on Bio-medical Engineering*.

[41] Hartikainen JEK, Tahvanainen KUO, Kuusela TA, Malik M (1998). Short-term measurement of heart rate variability. *Clinical Guide to Cardiac Autonomic Tests*.

[42] Lucreziotti S, Gavazzi A, Scelsi L, Inserra C, Klersy C, Campana C (2000). Five-minute recording of heart rate variability in severe chronic heart failure: correlates with right ventricular function and prognostic implications. *American Heart Journal*.

[43] Schroeder EB, Whitsel EA, Evans GW, Prineas RJ, Chambless LE, Heiss G (2004). Repeatability of heart rate variability measures. *Journal of Electrocardiology*.

[44] Nussinovitch U, Elishkevitz KP, Katz K, Nussinovitch M, Segev S, Volovitz B (2011). Reliability of Ultra-Short ECG Indices for Heart Rate Variability. *Annals of Noninvasive Electrocardiology: the Official Journal of the International Society for Holter and Noninvasive Electrocardiology, Inc*.

[45] Giardino ND, Lehrer PM, Edelberg R (2002). Comparison of finger plethysmograph to ECG in the measurement of heart rate variability. *Psychophysiology*.

[46] Jeyhani V, Mahdiani S, Peltokangas M, Vehkaoja A (2015). Comparison of HRV parameters derived from photoplethysmography and electrocardiography signals. *Annual International Conference of the IEEE Engineering in Medicine and Biology Society. IEEE Engineering in Medicine and Biology Society. Annual International Conference*.

[47] Malik M, Camm AJ (1990). Heart rate variability. *Clinical Cardiology*.

[48] Tegegne BS, Man T, van Roon AM, Riese H, Snieder H (2018). Determinants of heart rate variability in the general population: The Lifelines Cohort Study. *Heart Rhythm*.

[49] Malik M, Cripps T, Farrell T, Camm AJ (1989). Prognostic value of heart rate variability after myocardial infarction. A comparison of different data-processing methods. *Medical & Biological Engineering & Computing*.

[50] Malik M, Farrell T, Cripps T, Camm AJ (1989). Heart rate variability in relation to prognosis after myocardial infarction: selection of optimal processing techniques. *European Heart Journal*.

[51] Wijbenga JA, Balk AH, Meij SH, Simoons ML, Malik M (1998). Heart rate variability index in congestive heart failure: relation to clinical variables and prognosis. *European Heart Journal*.

[52] Malik M, Padmanabhan V, Olson WH (1999). Automatic measurement of long-term heart rate variability by implanted single-chamber devices. *Medical & Biological Engineering & Computing*.

[53] Malik M, Hnatkova K, Huikuri HV, Lombardi F, Schmidt G, Zabel M (2019). Rebuttal from Marek Malik, Katerina Hnatkova, Heikki V. *The Journal of Physiology*.

[54] Soejima K, Akaishi M, Meguro T, Oyamada K, Yoshikawa T, Mitamura H (2000). Age-adjusted heart rate variability as an index of the severity and prognosis of heart failure. *Japanese Circulation Journal*.

[55] Malik M, Camm AJ (1993). Components of heart rate variability–what they really mean and what we really measure. *The American Journal of Cardiology*.

[56] Pomeranz B, Macaulay RJ, Caudill MA, Kutz I, Adam D, Gordon D (1985). Assessment of autonomic function in humans by heart rate spectral analysis. *The American Journal of Physiology*.

[57] Billman GE, Huikuri HV, Sacha J, Trimmel K (2015). An introduction to heart rate variability: methodological considerations and clinical applications. *Frontiers in Physiology*.

[58] Hopf HB, Skyschally A, Heusch G, Peters J (1995). Low-frequency spectral power of heart rate variability is not a specific marker of cardiac sympathetic modulation. *Anesthesiology*.

[59] Notarius CF, Floras JS (2001). Limitations of the use of spectral analysis of heart rate variability for the estimation of cardiac sympathetic activity in heart failure. *Europace: European Pacing, Arrhythmias, and Cardiac Electrophysiology: Journal of the Working Groups on Cardiac Pacing, Arrhythmias, and Cardiac Cellular Electrophysiology of the European Society of Cardiology*.

[60] Dantas EM, Sant’Anna ML, Andreão RV, Gonçalves CP, Morra EA, Baldo MP (2012). Spectral analysis of heart rate variability with the autoregressive method: what model order to choose?. *Computers in Biology and Medicine*.

[61] Xhyheri B, Manfrini O, Mazzolini M, Pizzi C, Bugiardini R (2012). Heart rate variability today. *Progress in Cardiovascular Diseases*.

[62] Malik M, Hnatkova K, Huikuri HV, Lombardi F, Schmidt G, Zabel M (2019). CrossTalk proposal: Heart rate variability is a valid measure of cardiac autonomic responsiveness. *The Journal of Physiology*.

[63] Billman GE (2013). The LF/HF ratio does not accurately measure cardiac sympatho-vagal balance. *Frontiers in Physiology*.

[64] Furlan R, Guzzetti S, Crivellaro W, Dassi S, Tinelli M, Baselli G (1990). Continuous 24-hour assessment of the neural regulation of systemic arterial pressure and RR variabilities in ambulant subjects. *Circulation*.

[65] Kuusela T, Kamath MV (2012). Methodological aspects of heart rate variability analysis. *In heart rate variability (HRV) signal analysis*.

[66] Deka B, Deka D (2023). Nonlinear analysis of heart rate variability signals in meditative state: a review and perspective. *Biomedical Engineering Online*.

[67] Hnatkova K, Copie X, Staunton A, Malik M (1995). Numeric processing of Lorenz plots of R-R intervals from long-term ECGs. Comparison with time-domain measures of heart rate variability for risk stratification after myocardial infarction. *Journal of Electrocardiology*.

[68] Huikuri HV, Mäkikallio TH, Perkiömäki J (2003). Measurement of heart rate variability by methods based on nonlinear dynamics. *Journal of Electrocardiology*.

[69] Mahon NG, Hedman AE, Padula M, Gang Y, Savelieva I, Waktare JEP (2002). Fractal correlation properties of R-R interval dynamics in asymptomatic relatives of patients with dilated cardiomyopathy. *European Journal of Heart Failure*.

[70] Hoshi RA, Pastre CM, Vanderlei LCM, Godoy MF (2013). Poincaré plot indexes of heart rate variability: relationships with other nonlinear variables. *Autonomic Neuroscience: Basic & Clinical*.

[71] Porta A, Tobaldini E, Guzzetti S, Furlan R, Montano N, Gnecchi-Ruscone T (2007). Assessment of cardiac autonomic modulation during graded head-up tilt by symbolic analysis of heart rate variability. *American Journal of Physiology. Heart and Circulatory Physiology*.

[72] Koutelou M, Katsikis A, Flevari P, Theodorakis G, Livanis E, Georgiadis M (2009). Predictive value of cardiac autonomic indexes and MIBG washout in ICD recipients with mild to moderate heart failure. *Annals of Nuclear Medicine*.

[73] Shaffer F, Shearman S, Meehan ZM (2016). The promise of ultra-short-term (UST) heart rate variability measurements. *Biofeedback*.

[74] Scalvini S, Volterrani M, Zanelli E, Pagani M, Mazzuero G, Coats AJ (1998). Is heart rate variability a reliable method to assess autonomic modulation in left ventricular dysfunction and heart failure? Assessment of autonomic modulation with heart rate variability. *International Journal of Cardiology*.

[75] Vrtovec B, Okrajsek R, Golicnik A, Ferjan M, Starc V, Schlegel TT (2008). Atorvastatin therapy may reduce the incidence of sudden cardiac death in patients with advanced chronic heart failure. *Journal of Cardiac Failure*.

[76] Mikuz U, Poglajen G, Fister M, Starc V, Wu JC, Hsia H (2014). The presence of electromechanical mismatch in nonischemic dilated cardiomyopathy is associated with ventricular repolarization instability. *Journal of Cardiac Failure*.

[77] Arora R, Krummerman A, Vijayaraman P, Rosengarten M, Suryadevara V, Lejemtel T (2004). Heart rate variability and diastolic heart failure. *Pacing and Clinical Electrophysiology: PACE*.

[78] Guzzetti S, Mezzetti S, Magatelli R, Porta A, De Angelis G, Rovelli G (2000). Linear and non-linear 24 h heart rate variability in chronic heart failure. *Autonomic Neuroscience: Basic & Clinical*.

[79] Murray DR (2003). What is “heart rate variability” and is it blunted by tumor necrosis factor?. *Chest*.

[80] Landolina M, Gasparini M, Lunati M, Santini M, Rordorf R, Vincenti A (2008). Heart rate variability monitored by the implanted device predicts response to CRT and long-term clinical outcome in patients with advanced heart failure. *European Journal of Heart Failure*.

[81] Cheng C, Jiang J, Chen K, Hua W, Su Y, Xu W (2023). Device-evaluated autonomic nervous function for predicting ventricular arrhythmias and all-cause mortality in patients who underwent cardiac resynchronization therapy-defibrillator. *Frontiers in Physiology*.

[82] Sanderson JE, Yeung LY, Yeung DT, Kay RL, Tomlinson B, Critchley JA (1996). Impact of changes in respiratory frequency and posture on power spectral analysis of heart rate and systolic blood pressure variability in normal subjects and patients with heart failure. *Clinical Science (London, England: 1979)*.

[83] Guzzetti S, Cogliati C, Turiel M, Crema C, Lombardi F, Malliani A (1995). Sympathetic predominance followed by functional denervation in the progression of chronic heart failure. *European Heart Journal*.

[84] Bonaduce D, Petretta M, Marciano F, Vicario ML, Apicella C, Rao MA (1999). Independent and incremental prognostic value of heart rate variability in patients with chronic heart failure. *American Heart Journal*.

[85] Malliani A, Pagani M, Lombardi F, Cerutti S (1991). Cardiovascular neural regulation explored in the frequency domain. *Circulation*.

[86] Bigger JT, Fleiss JL, Steinman RC, Rolnitzky LM, Kleiger RE, Rottman JN (1992). Frequency domain measures of heart period variability and mortality after myocardial infarction. *Circulation*.

[87] Hadase M, Azuma A, Zen K, Asada S, Kawasaki T, Kamitani T (2004). Very low frequency power of heart rate variability is a powerful predictor of clinical prognosis in patients with congestive heart failure. *Circulation Journal: Official Journal of the Japanese Circulation Society*.

[88] Yamada T, Shimonagata T, Fukunami M, Kumagai K, Ogita H, Hirata A (2003). Comparison of the prognostic value of cardiac iodine-123 metaiodobenzylguanidine imaging and heart rate variability in patients with chronic heart failure: a prospective study. *Journal of the American College of Cardiology*.

[89] Tateishi O, Shouda T, Honda Y, Sakai T, Mochizuki S, Machida K (2002). Apnea-related heart rate variability and its clinical utility in congestive heart failure outpatients. *Annals of Noninvasive Electrocardiology: the Official Journal of the International Society for Holter and Noninvasive Electrocardiology, Inc*.

[90] McDonagh TA, Metra M, Adamo M, Gardner RS, Baumbach A, Böhm M (2021). 2021 ESC Guidelines for the diagnosis and treatment of acute and chronic heart failure. *European Heart Journal*.

[91] Moore RKG, Groves DG, Barlow PE, Fox KAA, Shah A, Nolan J (2006). Heart rate turbulence and death due to cardiac decompensation in patients with chronic heart failure. *European Journal of Heart Failure*.

[92] Galinier M, Pathak A, Fourcade J, Androdias C, Curnier D, Varnous S (2000). Depressed low frequency power of heart rate variability as an independent predictor of sudden death in chronic heart failure. *European Heart Journal*.

[93] Folino AF, Tokajuk B, Porta A, Romano S, Cerutti S, Volta SD (2005). Autonomic modulation and clinical outcome in patients with chronic heart failure. *International Journal of Cardiology*.

[94] Szabó BM, van Veldhuisen DJ, van der Veer N, Brouwer J, De Graeff PA, Crijns HJ (1997). Prognostic value of heart rate variability in chronic congestive heart failure secondary to idiopathic or ischemic dilated cardiomyopathy. *The American Journal of Cardiology*.

[95] Ponikowski P, Anker SD, Chua TP, Szelemej R, Piepoli M, Adamopoulos S (1997). Depressed heart rate variability as an independent predictor of death in chronic congestive heart failure secondary to ischemic or idiopathic dilated cardiomyopathy. *The American Journal of Cardiology*.

[96] Cygankiewicz I, Zareba W, Vazquez R, Bayes-Genis A, Pascual D, Macaya C (2009). Risk stratification of mortality in patients with heart failure and left ventricular ejection fraction >35%. *The American Journal of Cardiology*.

[97] Sredniawa B, Cebula S, Kowalczyk J, Batchvarov VN, Musialik-Lydka A, Sliwinska A (2010). Heart rate turbulence for prediction of heart transplantation and mortality in chronic heart failure. *Annals of Noninvasive Electrocardiology: the Official Journal of the International Society for Holter and Noninvasive Electrocardiology, Inc.*.

[98] Nolan J, Batin PD, Andrews R, Lindsay SJ, Brooksby P, Mullen M (1998). Prospective study of heart rate variability and mortality in chronic heart failure: results of the United Kingdom heart failure evaluation and assessment of risk trial (UK-heart). *Circulation*.

[99] Tamaki S, Yamada T, Okuyama Y, Morita T, Sanada S, Tsukamoto Y (2009). Cardiac iodine-123 metaiodobenzylguanidine imaging predicts sudden cardiac death independently of left ventricular ejection fraction in patients with chronic heart failure and left ventricular systolic dysfunction: results from a comparative study with signal-averaged electrocardiogram, heart rate variability, and QT dispersion. *Journal of the American College of Cardiology*.

[100] Smilde TDJ, van Veldhuisen DJ, van den Berg MP (2009). Prognostic value of heart rate variability and ventricular arrhythmias during 13-year follow-up in patients with mild to moderate heart failure. *Clinical Research in Cardiology: Official Journal of the German Cardiac Society*.

[101] Fauchier L, Babuty D, Cosnay P, Fauchier JP (1999). Prognostic value of heart rate variability for sudden death and major arrhythmic events in patients with idiopathic dilated cardiomyopathy. *Journal of the American College of Cardiology*.

[102] Anastasiou-Nana MI, Terrovitis JV, Athanasoulis T, Karaloizos L, Geramoutsos A, Pappa L (2005). Prognostic value of iodine-123-metaiodobenzylguanidine myocardial uptake and heart rate variability in chronic congestive heart failure secondary to ischemic or idiopathic dilated cardiomyopathy. *The American Journal of Cardiology*.

[103] Krüger C, Lahm T, Zugck C, Kell R, Schellberg D, Schweizer MWF (2002). Heart rate variability enhances the prognostic value of established parameters in patients with congestive heart failure. *Zeitschrift Fur Kardiologie*.

[104] Jiang W, Hathaway WR, McNulty S, Larsen RL, Hansley KL, Zhang Y (1997). Ability of heart rate variability to predict prognosis in patients with advanced congestive heart failure. *The American Journal of Cardiology*.

[105] Kaufmann DK, Raczak G, Szwoch M, Wabich E, Świątczak M, Daniłowicz-Szymanowicz L (2022). Baroreflex sensitivity but not microvolt T-wave alternans can predict major adverse cardiac events in ischemic heart failure. *Cardiology Journal*.

[106] Hashimoto H, Nakanishi R, Mizumura S, Hashimoto Y, Okamura Y, Yamanaka K (2020). Prognostic values of 123I-MIBG myocardial scintigraphy and heart rate variability in patients with heart failure with preserved ejection fraction. *Journal of Nuclear Cardiology: Official Publication of the American Society of Nuclear Cardiology*.

[107] Fauchier L, Marie O, Casset-Senon D, Babuty D, Cosnay P, Fauchier JP (2003). Ventricular dyssynchrony and risk markers of ventricular arrhythmias in nonischemic dilated cardiomyopathy: a study with phase analysis of angioscintigraphy. *Pacing and Clinical Electrophysiology: PACE*.

[108] Shah SA, Kambur T, Chan C, Herrington DM, Liu K, Shah SJ (2013). Relation of short-term heart rate variability to incident heart failure (from the Multi-Ethnic Study of Atherosclerosis). *The American Journal of Cardiology*.

[109] Stevenson WG, Epstein LM (2003). Predicting sudden death risk for heart failure patients in the implantable cardioverter-defibrillator age. *Circulation*.

[110] Soylu MO, Altun I, Basaran O, Uzun Y, Dogan V, Ergun G (2016). Impact of QRS morphology on heart rate turbulence and heart rate variability after cardiac resynchronization therapy in patients with heart failure. *European Review for Medical and Pharmacological Sciences*.

[111] Fantoni C, Raffa S, Regoli F, Giraldi F, La Rovere MT, Prentice J (2005). Cardiac resynchronization therapy improves heart rate profile and heart rate variability of patients with moderate to severe heart failure. *Journal of the American College of Cardiology*.

[112] Marijon E, Boveda S, Chevalier P, Bulava A, Winter JB, Lambiez M (2010). Monitoring of heart rate variability in heart failure patients with cardiac resynchronisation therapy: interest of continuous and didactic algorithm. *International Journal of Cardiology*.

[113] Gilliam FR, Singh JP, Mullin CM, McGuire M, Chase KJ (2007). Prognostic value of heart rate variability footprint and standard deviation of average 5-minute intrinsic R-R intervals for mortality in cardiac resynchronization therapy patients. *Journal of Electrocardiology*.

[114] Maisel WH, Stevenson LW (2003). Atrial fibrillation in heart failure: epidemiology, pathophysiology, and rationale for therapy. *The American Journal of Cardiology*.

[115] Myers G, Workman M, Birkett C, Ferguson D, Kienzle M (1992). Problems in measuring heart rate variability of patients with congestive heart failure. *Journal of Electrocardiology*.

[116] Schmidt G, Malik M, Barthel P, Schneider R, Ulm K, Rolnitzky L (1999). Heart-rate turbulence after ventricular premature beats as a predictor of mortality after acute myocardial infarction. *Lancet (London, England)*.

[117] Bauer A, Malik M, Schmidt G, Barthel P, Bonnemeier H, Cygankiewicz I (2008). Heart rate turbulence: standards of measurement, physiological interpretation, and clinical use: International Society for Holter and Noninvasive Electrophysiology Consensus. *Journal of the American College of Cardiology*.

[118] Forte G, Favieri F, Casagrande M (2019). Heart Rate Variability and Cognitive Function: A Systematic Review. *Frontiers in Neuroscience*.

[119] Nunan D, Sandercock GRH, George RS, Jakovljevic DG, Donovan G, Bougard R (2013). Cardiovascular autonomic control in patients undergoing left ventricular assist device (LVAD) support and pharmacologic therapy. *International Journal of Cardiology*.

[120] Murad K, Brubaker PH, Fitzgerald DM, Morgan TM, Goff DC, Soliman EZ (2012). Exercise training improves heart rate variability in older patients with heart failure: a randomized, controlled, single-blinded trial. *Congestive Heart Failure (Greenwich, Conn.)*.

[121] Ponikowski P, Piepoli M, Chua TP, Banasiak W, Francis D, Anker SD (1999). The impact of cachexia on cardiorespiratory reflex control in chronic heart failure. *European Heart Journal*.

[122] Rydlewska A, Maj J, Katkowski B, Biel B, Ponikowska B, Banasiak W (2013). Circulating testosterone and estradiol, autonomic balance and baroreflex sensitivity in middle-aged and elderly men with heart failure. *The Aging Male: the Official Journal of the International Society for the Study of the Aging Male*.

[123] Walker AM, Patel PA, Rajwani A, Groves D, Denby C, Kearney L (2016). Diabetes mellitus is associated with adverse structural and functional cardiac remodelling in chronic heart failure with reduced ejection fraction. *Diabetes & Vascular Disease Research*.

[124] Boveda S, Galinier M, Pathak A, Fourcade J, Dongay B, Benchendikh D (2001). Prognostic value of heart rate variability in time domain analysis in congestive heart failure. *Journal of Interventional Cardiac Electrophysiology: an International Journal of Arrhythmias and Pacing*.

[125] Ryan TJ, Anderson JL, Antman EM, Braniff BA, Brooks NH, Califf RM (1996). ACC/AHA guidelines for the management of patients with acute myocardial infarction: executive summary. A report of the American College of Cardiology/American Heart Association Task Force on Practice Guidelines (Committee on Management of Acute Myocardial Infarction). *Circulation*.

[126] Antman EM, Anbe DT, Armstrong PW, Bates ER, Green LA, Hand M (2004). ACC/AHA guidelines for the management of patients with ST-elevation myocardial infarction-executive summary: a report of the American College of Cardiology/American Heart Association Task Force on Practice Guidelines (Writing Committee to Revise the 1999 Guidelines for the Management of Patients With Acute Myocardial Infarction). *Circulation*.

[127] Kocaman SA, Taçoy G, Ozdemir M, Açıkgöz SK, Cengel A (2010). The preserved autonomic functions may provide the asymptomatic clinical status in heart failure despite advanced left ventricular systolic dysfunction. *Anadolu Kardiyoloji Dergisi: AKD = the Anatolian Journal of Cardiology*.

[128] Garet M, Degache F, Pichot V, Duverney D, Costes F, DA Costa A (2005). Relationship between daily physical activity and ANS activity in patients with CHF. *Medicine and Science in Sports and Exercise*.

[129] Kienzle MG, Ferguson DW, Birkett CL, Myers GA, Berg WJ, Mariano DJ (1992). Clinical, hemodynamic and sympathetic neural correlates of heart rate variability in congestive heart failure. *The American Journal of Cardiology*.

[130] Lombardi F, Huikuri H, Schmidt G, Malik M, e-Rhythm Study Group of EHRA (2018). The decline of rate and mortality of acute myocardial infarction. Almost there, still a long way to go. *European Journal of Preventive Cardiology*.

[131] Lombardi F, Huikuri H, Schmidt G, Malik M, e-Rhythm Study Group of European Heart Rhythm Association (2018). Short-term heart rate variability: Easy to measure, difficult to interpret. *Heart Rhythm*.

[132] Huikuri HV, Zabel M, Lombardi F, Malik M, e-Health, Digital Rhythm Study Group of the European Heart Rhythm Association (2017). Measurement of cardiovascular autonomic function: Where to go from here?. *International Journal of Cardiology*.

[133] Armoundas AA, Narayan SM, Arnett DK, Spector-Bagdady K, Bennett DA, Celi LA (2024). Use of Artificial Intelligence in Improving Outcomes in Heart Disease: A Scientific Statement From the American Heart Association. *Circulation*.

[134] Holmstrom L, Chugh H, Nakamura K, Bhanji Z, Seifer M, Uy-Evanado A (2024). An ECG-based artificial intelligence model for assessment of sudden cardiac death risk. *Communications Medicine*.

[135] Sau A, Ribeiro AH, McGurk KA, Pastika L, Bajaj N, Gurnani M (2024). Prognostic Significance and Associations of Neural Network-Derived Electrocardiographic Features. *Circulation. Cardiovascular Quality and Outcomes*.

[136] Malik M (2024). The value of invisible electrocardiography. *Heart Rhythm*.

[137] Hnatkova K, Andršová I, Novotný T, Britton A, Shipley M, Vandenberk B (2022). QRS micro-fragmentation as a mortality predictor. *European Heart Journal*.

[138] Kadhiresan K, Carlson G (2004). The role of implantable sensors for management of heart failure. *Studies in Health Technology and Informatics*.

[139] Adamson PB, Smith AL, Abraham WT, Kleckner KJ, Stadler RW, Shih A (2004). Continuous autonomic assessment in patients with symptomatic heart failure: prognostic value of heart rate variability measured by an implanted cardiac resynchronization device. *Circulation*.

[140] Scholte NTB, van Ravensberg AE, Shakoor A, Boersma E, Ronner E, de Boer RA (2024). A scoping review on advancements in noninvasive wearable technology for heart failure management. *NPJ Digital Medicine*.

[141] Guzik P, Malik M (2016). ECG by mobile technologies. *Journal of Electrocardiology*.

[142] Cao R, Azimi I, Sarhaddi F, Niela-Vilen H, Axelin A, Liljeberg P (2022). Accuracy Assessment of Oura Ring Nocturnal Heart Rate and Heart Rate Variability in Comparison With Electrocardiography in Time and Frequency Domains: Comprehensive Analysis. *Journal of Medical Internet Research*.

[143] Helánová K, Šišáková M, Hnatkova K, Novotný T, Andršová I, Malik M (2024). Development of autonomic heart rate modulations during childhood and adolescence. *Pflugers Archiv: European Journal of Physiology*.

[144] Šišáková M, Helánová K, Hnatkova K, Andršová I, Novotný T, Malik M (2024). Intra-Individual Relationship between Heart Rate Variability and the Underlying Heart Rate in Children and Adolescents. *Journal of Clinical Medicine*.

[145] Šišáková M, Helánová K, Hnatkova K, Andršová I, Novotný T, Malik M (2024). Speed of heart rate changes during postural provocations in children and adolescents. *Scientific Reports*.

